# Valorization of milling byproducts and ergot-sclerotia-contaminated rye via clostridial ABE fermentation

**DOI:** 10.1186/s13068-024-02590-6

**Published:** 2024-11-30

**Authors:** Holger Edelmann, Nils Thieme, Armin Ehrenreich, Vladimir Zverlov, Wolfgang Liebl

**Affiliations:** 1 Chair of Microbiology, Technical University of Munich, Emil-Ramann-Str. 4, 85354 Freising, Germany; 2 Present Address: Planet A Foods GmbH, Fraunhoferstrasse 11A, 82152 Planegg, Germany

**Keywords:** ABE fermentation, Butanol, Clostridia, Milling byproducts, Ergot sclerotia

## Abstract

**Background:**

Acetone–butanol–ethanol (ABE) fermentation by solventogenic clostridia can be harnessed to produce CO_2_ emission neutral bio-based 1-butanol, a valuable compound with a broad range of applications, e.g., in industrial production, as a solvent and as a fuel additive or replacement. However, the relatively low butanol titers and high feedstock costs prevent bio-butanol production on an industrial scale. Agricultural side-stream materials, like milling byproducts, are starch-rich, low-cost and produced all year round. They could be suitable substrates for bio-butanol production by ABE fermentation.

**Results:**

The milling byproducts wheat red dog (WRD), rye second flour (RSF), wheat bran (WB), rye bran (RB) and ergot sclerotia-containing rye waste stream (ER) were found to contain between ~ 30 and ~ 85% glucan, most of which was starch based. WRD, RSF and ER had the highest glucan content, while the brans contained significant xylan concentrations. Four strains selected from the collection of solventogenic clostridia available in our group produced > 6 g/L butanol on the majority of these substrates, with *Clostridium beijerinckii* NCIMB 8052 showing the best ABE production performance when regarding all tested substrates. Toxic ergot sclerotia-containing waste material was found to be a suited substrate for ABE fermentation. Strain NCIMB 8052 exhibited butanol titers of up to 9 g/L on substrate mixtures of WRD plus ER and the highest butanol yield per used sugars. Finally, a semi-continuous ABE fermentation of *C. beijerinckii* NCIMB 8052 on WRD plus ER could be maintained for 96 h. The volumetric ABE productivity during the continuous phase of fermentation was ~ 0.41 g L^−1^ h^−1^ and a total of 37.7 g ABE was produced out of 168.2 g substrate.

**Conclusions:**

Based on their carbohydrate composition, WRD, RSF and ER were the milling byproducts best suited as substrates for bio-butanol production by clostridial ABE fermentation. Importantly, also ergot sclerotia-containing waste materials can be used as substrates, which can help to reduce process costs. The semi-continuous fermentation showed that clostridial ABE fermentation on milling byproducts may represent a suitable avenue for commercial butanol production after further process and/or strain optimization.

**Supplementary Information:**

The online version contains supplementary material available at 10.1186/s13068-024-02590-6.

## Introduction

As greenhouse gas emissions of anthropogenic origin are major contributors to climate change [1], the need to replace fossil fuels with more eco-friendly alternatives increases with every year [2, 3]. Especially biofuels and other biorefinery-based platform chemicals, such as 1-butanol, have the potential to relieve the pressure on the climate by human made carbon emissions [4].

1-Butanol is used as solvent in the production in coatings, painting and cleaning products as well as of plasticizers, lubricants as well as intermediate in chemical syntheses [4, 5]. Due to its high energy density, reduced hygroscopy and chemical properties, which are comparable to diesel or gasoline, butanol can be used as fuel additive or replacement [6, 7]. Furthermore, blends of butanol and diesel exhibit reduced nitrogen oxide and carbon monoxide emissions [8].

Solventogenic clostridia can produce acetone–butanol–ethanol (ABE) through fermentation [9]. ABE fermentation is a bi-phasic process, where acetic and butyric acids are produced during the growth phase, followed by their re-assimilation and conversion into the respective solvents [9]. This process was already used on a large scale for butanol production in the Soviet Union, but the fermentation plants were closed in the 1960s, when cheaper chemical methods became available [10]. Still, by overcoming economically limiting factors, such as substrate cost, large-scale ABE fermentation plants might be lucrative and ecological alternatives for butanol production. Using agricultural side-stream materials, e.g., corncob residues, rice bran or milling byproducts, as substrate for ABE fermentation can significantly reduce operating costs of fermentation plants, while still allowing sufficient ABE productivity [11–13].

Milling byproducts are low-cost side-stream materials of the milling industry, which are produced all year round and account for ~ 25% to 30% of milling products [14]. So far, they are mostly used as animal feeds [14], but they contain a significant amount of starch and hemicelluloses, but almost no lignin or cellulose [15, 16]. This makes them suitable substrates for ABE fermentation, as most industrially relevant solventogenic clostridia can utilize the majority of plant derived mono-, di- and polysaccharides, e.g., starch, xylan or xyloglucan, as carbon source [17–19]. Furthermore, milling byproducts were recently evaluated as substrate for clostridial ABE fermentation. The economic analysis showed that small- to mid-scale fermentation plants can be profitable when wheat red dog (WRD) is utilized as substrate [13].

However, there are more milling byproducts besides WRD that could be used for ABE fermentation. Especially ergot sclerotia-containing rye waste-streams are currently not utilized, as they contain a variety of alkaloids, such as ergometrine, ergotamine and ergocryptine, which are toxic for animals and humans [20–23]. These can cause severe symptoms including convulsions, hallucinations, muscle spasms, nausea, vomiting, and peripheral vasoconstriction and burning sensation [24]. The pharmacological effects of these alkaloids are likely due to their structural similarity to human neurotransmitters such as noradrenaline, dopamine, and serotonin [25]. Effects on bacterial fermentation have not been investigated.

Ergot sclerotia are produced by *Claviceps* spp., which mostly infect rye, but also barley, oat and wheat [26–28]. Currently, ergot sclerotia are separated from rye during the milling process by optical cereal grain sorting techniques and later disposed of by burning [29, 30]. As these waste-streams have no economic value, but are rich in starch, they could be valorized using clostridial ABE fermentation.

In this study, we determined the sugar composition of the milling byproducts WRD, rye second flour (RSF), wheat bran (WB), rye bran (RB) and ergot sclerotia-containing rye (ER) waste. Based on these results, the usability of these materials as substrate for clostridial ABE fermentation was determined. To optimize the economics of ABE fermentation with milling byproducts, it was also of interest to evaluate the use of mixtures of WRD plus ER as substrates together with enzymatic pretreatment with (hemi)cellulases. A semi-continuous fermentation approach was used to test a mixture of selected milling byproducts for ABE productivity in order to assess if they could be suitable substrates for industrial scale fermentations.

## Results and discussion

### Polysaccharide composition of milling byproducts

**Fig. 1:**
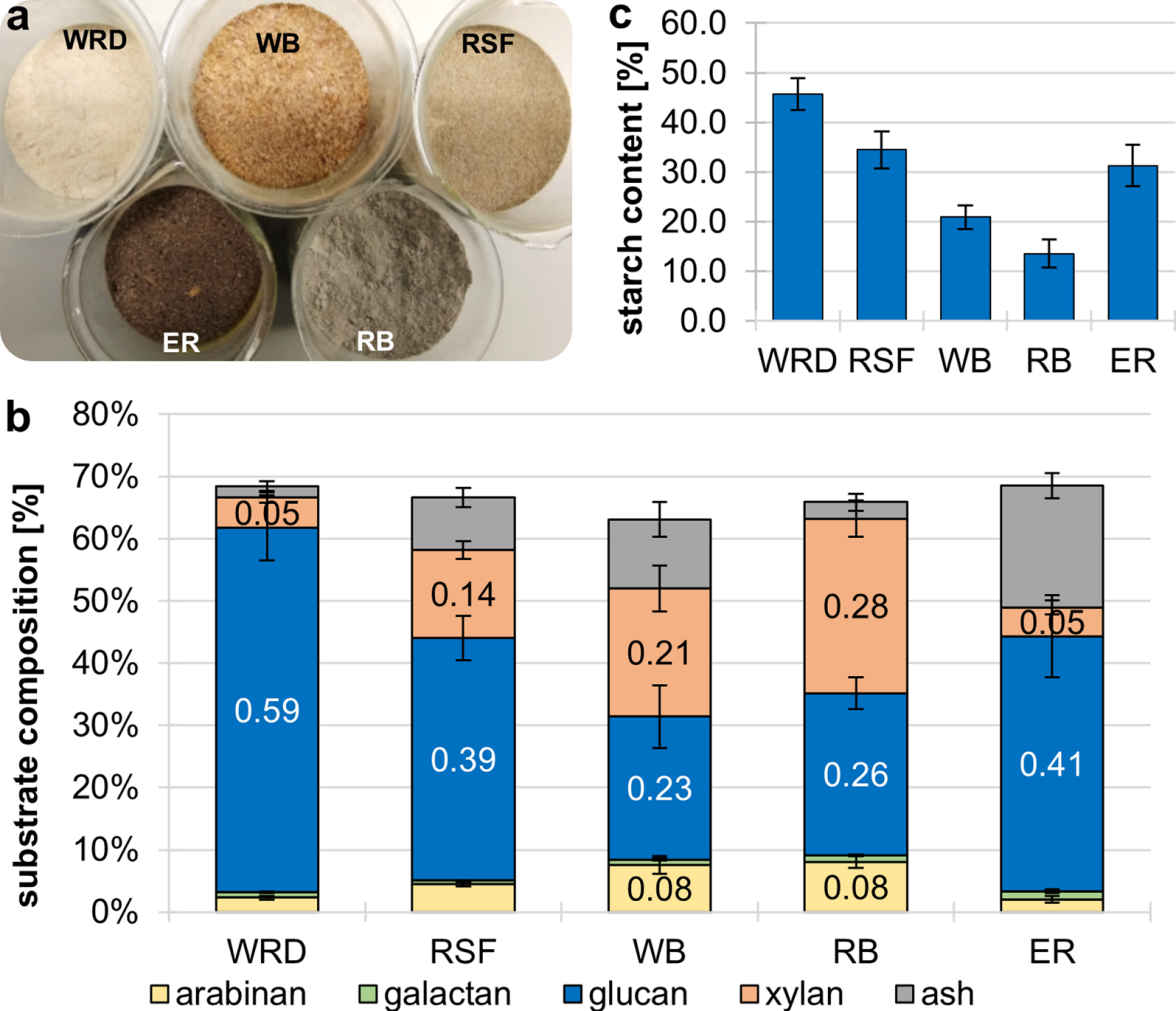
Compositional analysis of milling byproducts. **a** Texture of the used cereal grist. **b** Determination of main sugar contents of substrate by acidic hydrolysis. Monosaccharides were measured using HPAEC-PAD. Ash content was determined by dry weight. **c** Analysis of soluble starch content in the respective substrates. Starch content was determined through enzymatic digestion of the substrates followed by reducing end assay. Error bars represent standard deviation (N = 3)

Wheat red dog (WRD) and wheat middlings were analyzed as substrates for ABE fermentation in a previous study [13]. An economic analysis showed that WRD can be suitable for butanol production in small- to mid-scale fermentation plants. However, other milling byproducts may also be of interest for industrial ABE production, such as rye second flour (RSF), wheat bran (WB), rye bran (RB) and ergot sclerotia-containing rye (ER) (Fig. 1a).

To assess the suitability of these substrates (kindly supplied by Bayerischer Müllerbund e. V., Munich, Bavaria, Germany) for ABE fermentation, we first performed acidic hydrolysis of the biomass samples to determine their sugar composition (Fig. 1b). Only l-arabinose, d-galactose, d-glucose and d-xylose moieties were detected in this experiment.

The used milling byproducts show a total sugar content between 49 and 67% (WRD 67%, RSF 58%, WB 52%, RB 63%, ER 49%). WRD, RSF and ER showed the highest glucose content of the substrates (86, 59 and 60%, respectively). Additionally, RSF, WB and RB contained 21, 33 and 43% xylose, while WB and RB also contained significant amounts of arabinose (12% each). Expectedly, the bran substrates were richer in hemicellulose-typical pentose sugars, as they consist mostly of grain husk, while WRD, RSF and ER were rich in glucan. These data are in accordance with published carbohydrate composition of typical *Poaceae* species, such as *Triticum aestivum* (common wheat) and *Secale cereale* (rye), which contain mainly hemicelluloses formed of xylan backbones (20 to 50%) or mixed linkage glucans (10 to 30%, only present in primary cell wall), with little to no mannan content [15, 31]. Wheat and rye grains also contain larger fractions of hemicelluloses (rye: 9.3%, wheat: 8.2%) composed of xylose and arabinose, but they are mainly composed of 61 to 67% starch. However, they contain only a very small amount of cellulose (< 1.7%) and almost no crude lignin [31, 32].

To determine the amount of soluble starch that constitutes the majority of the glucan fraction of milling byproducts, we performed an enzymatic digest of the biomasses with a α-amylase and α-glucosidase mixture (Fig. 1c). Our analysis showed that starch content was high in WRD, RSF and ER, with soluble and enzymatically accessible starch contents between 30 and 45%, while both bran substrates contained only small amounts of soluble starch (between 13 and 20%). In literature, starch contents between 65 and 75% are described for WRD and RSF, while the brans contain only 10 to 25% starch [16, 33]. It is feasible, that the remaining ~ 30% glucan in WRD and RSF in our study are primarily composed of insoluble starch. Starch is composed of amylose and amylopectin, with differences in their structure and physicochemical properties. Amylopectin is branched and water-soluble, while amylose forms helical and overlapping chains, which make the polysaccharide insoluble in water and more recalcitrant [34–36].

Overall, the compositional analyses of the milling byproducts indicate a high suitability of WRD, RSF and ER for clostridial ABE fermentation, as they are rich in starch and contain small amounts of hemicellulose. Both starch and hemicelluloses can be utilized as carbon sources by certain clostridia strains [18].

### Milling byproducts as substrates for ABE fermentation

To determine which milling byproducts were suitable for clostridial ABE fermentation, we incubated four strains selected from the strain collection at the Chair of Microbiology (Technical University of Munich, Germany) on GM plus 15% of either WRD, RSF, WB, RB, or ER (Table 1). The fermentation products in the culture supernatant were measured with GC. The strains *Clostridium beijerinckii* NCIMB 8052 (CBEI), *C. saccharobutylicum* DSM13684 (CSAC), *C. diolis* DSM 15410 (CDIO) and *C. saccharoperbutylacetonicum* N1-504 (CSPA) were all able to utilize the substrates rich in starch WRD, RSF and ER and produced > 6 g/L butanol. Only *C. beijerinckii* NCIMB 8052 produced more than 6 g/L butanol on WB and RB. These results align with previous studies, where these strains also showed good ABE production on starch-rich substrates, but reduced solvent production on fiber- and hemicellulose-rich materials [13]. The reduced ABE production of the tested strains on the bran substrates could be caused by multiple factors. Firstly, starch is rich in glucose and a relatively easily macerated substrate [37], which can cause carbon catabolite repression (CCR). CCR describes the preference of organisms to utilize easily metabolizable carbohydrates, such as d-glucose, over polysaccharides, which require an increased energy and material expenditure on the side of the organism for degradation [38–40]. Also, the bran substrates contain a lower concentration of the easily degraded glucans compared to the other substrates. Secondly, hemicellulose is a more complex polysaccharide than starch, usually decorated with sugar side chains on the xylose backbone [41], and more enzyme activity types are required for its complete degradation [42, 43]. Furthermore, hemicellulase expression is usually downregulated during CCR inducing conditions [6, 44]. Hence, hemicellulose is most likely utilized after the depletion of most of the starch, which leads to delayed hemicellulase expression and less available carbon source for ABE fermentation. The starch-rich milling byproducts WRD, RSF and ER are therefore better suited as substrates for commercial ABE fermentation.

**Table 1:** Solvent production of clostridia strains on medium with milling byproducts as substrate

Substrate	strain	Solvent production [g/L]						
		Acetone			Butanol			Ethanol
15% WRD	*C. beijerinckii* NCIMB 8052	4.4	±	0.4	13.1	±	0.7	N. Q
	*C. diolis* DSM 15410	2.2	±	0.1	7.2	±	0.2	N. Q
	*C. saccharobutylicum* DSM 13864	6.4	±	0.8	12.8	±	1.9	N. Q
	*C. saccharoperbutylacetonicum* N1-504	7.0	±	2.8	10.0	±	4.0	N. Q
15% RSF	*C. beijerinckii* NCIMB 8052	5.3	±	0.4	13.5	±	1.0	N. Q
	*C. diolis* DSM 15410	5.2	±	0.6	13.3	±	1.9	N. Q
	*C. saccharobutylicum* DSM 13864	5.5	±	1.0	9.8	±	1.7	N. Q
	*C. saccharoperbutylacetonicum* N1-504	0.8	±	0.0	9.9	±	1.7	N. Q
15% WB	*C. beijerinckii* NCIMB 8052	1.4	±	0.0	7.1	±	0.9	N. Q
	*C. diolis* DSM 15410	1.3	±	0.1	4.1	±	0.1	N. Q
	*C. saccharobutylicum* DSM 13864	4.2	±	3.2	7.4	±	0.4	N. Q
	*C. saccharoperbutylacetonicum* N1-504	1.6	±	0.0	2.2	±	0.1	N. Q
15% RB	*C. beijerinckii* NCIMB 8052	3.5	±	0.6	9.9	±	0.2	N. Q
	*C. diolis* DSM 15410	1.6	±	0.1	5.4	±	0.6	N. Q
	*C. saccharobutylicum* DSM 13864	1.8	±	0.2	1.8	±	0.4	N. Q
	*C. saccharoperbutylacetonicum* N1-504	0.4	±	0.0	2.3	±	0.2	N. Q
15% ER	*C. beijerinckii* NCIMB 8052	3.3	±	0.1	11.1	±	0.4	N. Q
	*C. diolis* DSM 15410	3.7	±	0.2	11.3	±	0.6	N. Q
	*C. saccharobutylicum* DSM 13864	6.6	±	0.7	11.6	±	0.8	N. Q
	*C. saccharoperbutylacetonicum* N1-504	3.3	±	0.1	10.3	±	0.4	N. Q

The strains were incubated 72 h at 34 °C in GM plus 15% of respective carbon source. The fermentation products acetone and butanol were measured using GC-FID. Ethanol was found, but concentrations were low and not quantified (N.Q.). The experiment was performed in duplicate

*C. beijerinckii* NCIMB 8052 showed the best or second-best butanol production on all tested substrates and therefore was used as the main test strain in all following experiments.

**Fig. 2:**
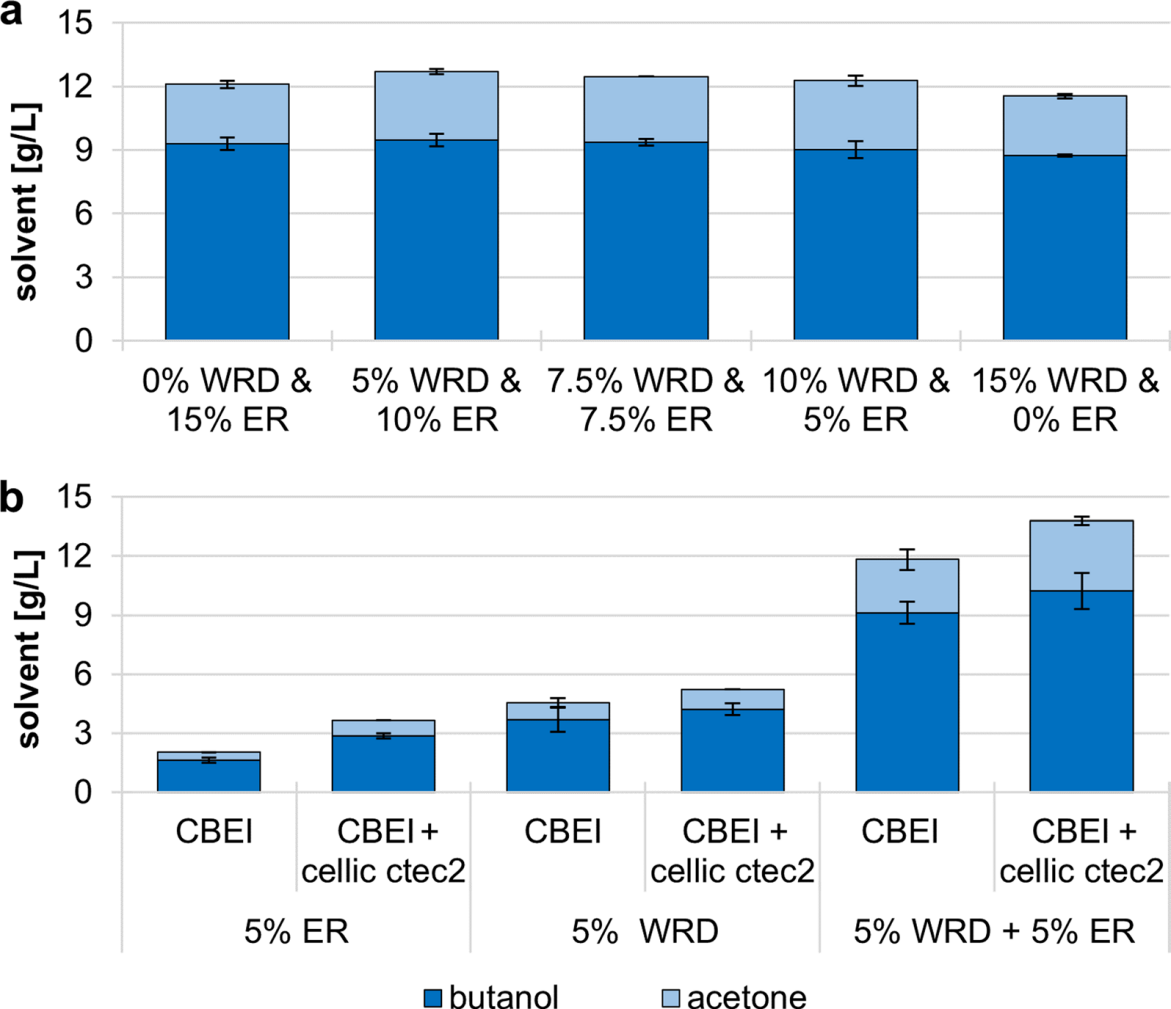
ABE production of *C. beijerinckii* incubated on WRD and ER mixtures or enzymatically pretreated substrates. **a** The strain was incubated over 72 h on GM with the mixtures of WRD and ER. 15% WRD and 15% ER were used as control conditions. **b** The substrates were enzymatically pretreated for 24 h at 50 °C with 0.5% (v/v) of the cellulase and hemicellulase mixture cellic ctec2. Afterwards, *C. beijerinckii* NCIMB 8052 was incubated on these substrates as described in a). Error bars represent standard deviation (N = 3)

Using mixtures of different milling byproducts may be a suited strategy to overcome material shortages in the future and could help to reduce feedstock costs of the fermentation process. Especially ER is suited as substrate supplementation, as it is produced in relatively low but in significant quantities, has no value on the market, but is rich in starch. Therefore, we tested the ABE production of *C. beijerinckii* NCIMB 8052 on mixtures of WRD and ER. The strain was incubated for 72 h in GM plus one of the following substrate mixes; 10% (w/v) WRD plus 5% (w/v) ER, 7.5% (w/v) WRD plus 7.5% (w/v) ER or 5% (w/v) WRD plus 10% (w/v) ER (Fig. 2a). Additionally, GM with 15% (w/v) WRD or 15% (w/v) ER were used as controls. All tested conditions showed a similar butanol production of ~ 9 g/L, including the controls. Mixing WRD with ER is therefore a suitable option to increase the available amount of fermentable substrate, as well as to keep substrate costs low. It also shows that there is no inhibition of the butanol production by the ergot sclerotia in the substrate.

Butanol production on complex substrates, e.g., corn stover, rice bran or milling byproducts, can be improved by (partially) hydrolyzing the substrates prior to fermentation [12, 13]. Dilute acid pretreatments are effective at macerating plant material and liberating soluble sugars [45], but prolong the whole process and are costly [46]. Recently, ionic liquids emerged as another route to deconstruct polysaccharides [47–49], but economic analyses of ABE fermentations using ionic liquid pretreated substrates were not performed to date. Enzymatic pretreatment of substrates can also be used to increase the amount of available soluble sugars in the fermentation broth [13, 45, 50]. We therefore determined if enzymatic pretreatment of ER, WRD or a mixture of both milling byproducts can significantly improve butanol production.

The commercially available cellulase and hemicellulase mixture cellic ctec2 was used to macerate 5% (w/v) ER, 5% (w/v) WRD or 5% (w/v) ER plus 5% (w/v) WRD in GM (Fig. 2b). The substrates were incubated for 24 h at 50 °C with 0.5% (v/v) enzyme mixture, followed by incubation with *C. beijerinckii* NCIMB 8052 for 72 h at 34 °C. Interestingly, the strain produced less than half the amount of butanol on ER (1.6 g/L) compared to WRD (3.7 g/L), which differed from the similar solvent production observed at a higher concentration (15%) of the substrates (Fig. 2a). Enzymatic pretreatment with cellic ctec2 improved butanol production by *C. beijerinckii* incubated on ER by 76% to 2.9 g/L, while butanol production on WRD was merely improved by 14% to 4.2 g/L. Butanol production of *C. beijerinckii* incubated on a mixture of 5% WRD and 5% ER on the other hand was not improved after enzymatic pretreatment. It appears that the combined amount of 10% (w/v) substrate contained sufficient starch for the strain to produce large quantities of butanol.

It is possible that the effects of the enzymatic hemicellulose degradation on ABE production is diminished by the abundance of starch in WRD, which likely causes CCR. Furthermore, the uptake of soluble sugars is also subject to a form of CCR, as most organisms prefer glucose over other monosaccharides [38, 40, 51]. This behavior leads to diauxic growth, prevents the simultaneous utilization of different sugars and abolishes the positive effects more readily available non-glucose sugars could have on ABE production [52].

Our data indicate that cellulase and hemicellulase pretreatment is not necessary for fermentations using high concentrations of starch-rich substrate. It is still possible that long-term (semi-)continuous fermentations benefit from an early depolymerization of hemicellulose, which might reduce the transition time of clostridia from starch-rich to low starch metabolism and extenuate losses in ABE production.

### Profiling substrate degradation and solvent production

To characterize the substrate degradation ability of different strains, we cultured *C. beijerinckii* NCIMB 8052 (CBEJ), *C. saccharobutylicum* DSM13684 (CSA) and *C. saccharoperbutylacetonicum* N1-504 (CSPA) in 100 ml serum flasks on GM with 5%, 10% and 15% substrate (w/v) at 34 °C for 120 h. The substrate consisted of a 2:1 ratio of WNM and ER. Polysaccharide degradation was determined by acid hydrolysis of 1.0 ml dry residue of used culture broth or culture broth prior to fermentation as reference. In unfermented broth, about 65% of the dry weight corresponded to the monosaccharides glucose, xylose, arabinose, galactose, which were set to 100% in following analysis.

**Fig. 3:**
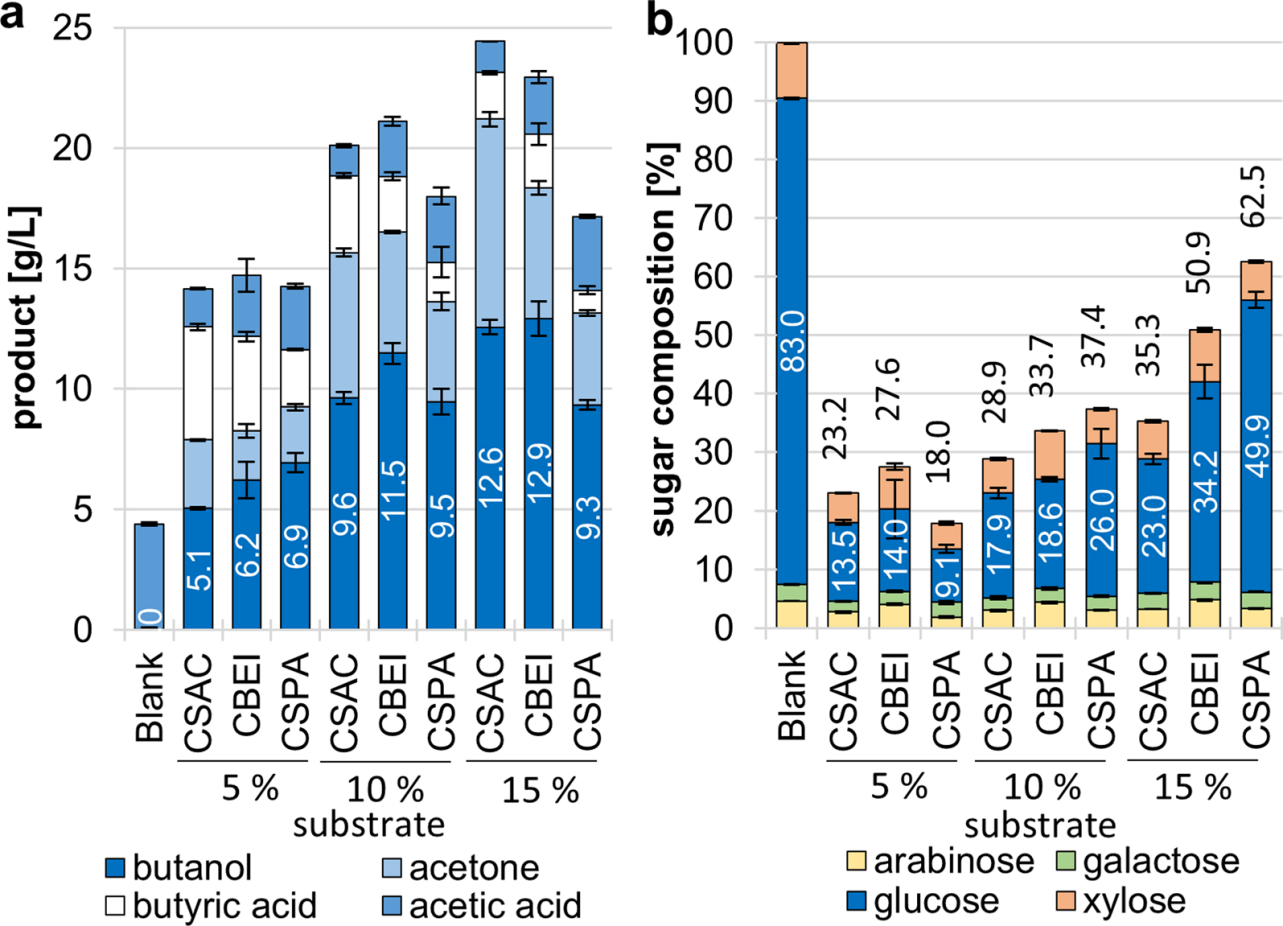
Substrate degradation profile different Clostridia. *C. beijerinckii* NCIMB 8052 (CBEJ), *C. saccharobutylicum* DSM 13864 (CSA) and *C. saccharoperbutylacetonicum* N1-504 (CSPA) were incubated in GM with a mixture of 10% WRD plus 5% ER as carbon source for 120 h at 34 °C**. a** The remaining polysaccharide composition of the used substrate was determined through acidic hydrolysis and measured with HPAEC-PAD. The total sugar residue in percent is given above the bars. **b** Product formation of this experiment was measured with GC-FID. (N = 3)

At 5% substrate load, the strains left only 18.0% (CSPA), 23.2% (CSAC) and 27.6% (CBEJ) of total sugars unfermented (Fig. 3b). The glucose fraction (82.96%), consisting mainly of starch and minor amounts of mixed linkage glycan (~ 3%) and cellulose (~ 2.5%) [31], was reduced to 9.1% (CSPA), 13.5% (CSAC) and 14.0% (CBEJ).

Xylose fractions were reduced from 9.51% of the total sugars to 4.4% (CSPA), 5.0% (CSAC) and 7.2% (CBEJ). The same pattern is followed by arabinose, as it is mostly found in the hemicellulose arabinoxylan.

All three tested strains were more efficient utilizing complex substrate at 5% substrate load. *C. saccharoperbutylacetonicum* achieved the best ratio of sugar utilization, the lowest butyrate titer, and the highest butanol titer of 6.9 g/L (Fig. 3a). This may be due to the strain's superior ability to reutilize formed acids and has been described previously [53]. Under this condition, the best substrate productivity of all strains for butanol of 0.136 g/g substrate was achieved with *C. saccharoperbutylacetonicum* (Fig. S1).

Higher substrate loads reduced degradation performance of all strains. *C. saccharoperbutylacetonicum* leaves significantly more sugars at 10% substrate load, while *C. beijerinckii* shows a strong increase at 15% load. *C. saccharobutylicum* is the least affected and can still degrade substrate to 35.3% residual sugars at 15% load. The tolerance of *C. saccharobutylicum* to high substrate loads and butanol titers is described in the literature for molasses fermentation [54, 55]. At 15% load *C. saccharobutylicum* and *C. beijerinckii* produced up to 12.6 g/L and 12.9 g/L butanol, which is close to the butanol production limit of native ABE-producing strains [56, 57]. In this of concentration range, butanol is toxic and inhibits further cell growth [58, 59].

Although *C. beijerinckii* achieved a slightly lower substrate degradation than *C. saccharobutylicum,* it showed a superior butanol yield per substrate and per sugar used in all fermentations, which was 0.255 g/g and 0.115 g/g at 10% substrate loading, respectively. The strain was therefore used in further experiments. The butanol titer and yield per used sugars are comparable with conventional batch fermentations on glucose, only the amount of residual sugars is higher since here complex substrate was used [56, 60].

In general, higher butanol titers from batch fermentations are desirable to reduce extraction costs. Here, the substrate load is a critical parameter and must be balanced to achieve high butanol yields with high substrate utilization.

These data will help to find and optimize a suitable production process. Another way to regulate the process and cope with catabolite inhibition is to use different fermentation modes such as fed-batch or continuous fermentation processes [61]

### Effect and fate of ergot alkaloids in clostridial fermentation

The ergot-sclerotia-contaminated substrate (ER) used in this study contained high levels of sclerotia, approximately 30% ergot sclerotia. We demonstrated that both pure ER substrate and mixtures with WRD resulted in similar butanol titers when fermented by *C. beijerinckii*, with no negative effects observed (Fig. 2). In addition, other strains of solventogenic clostridia were able to ferment pure ergot-sclerotia-contaminated rye to high butanol titers of 10.3 to 11.6 g/L (Table 1). Small differences in total butanol from the fermentation of pure ER compared to WRD are rather explained by the lower sugar content. WRD and ER contain 67% and 49% sugars, respectively (Fig. 1). Our experiments show that ergot alkaloids have no significant effect on fermentation and that contaminated substrate can be successfully converted to solvents.

The release of ergot alkaloids in the fermentation broth and later into the extracted products must be considered as risk factor when utilizing ER as substrate. The need to remove alkaloids would also increase post-fermentation processing costs. To this end, using ErgoREAD ELISA assays we determined the ergot alkaloid concentration in the ER substrate, the fermentation broth before and after fermentation, as well as after extraction of ABE from culture supernatant. *C. beijerinckii* NCIMB 8052 was grown on GM with 15% (w/v) ER for these experiments.

**Fig. 4:**
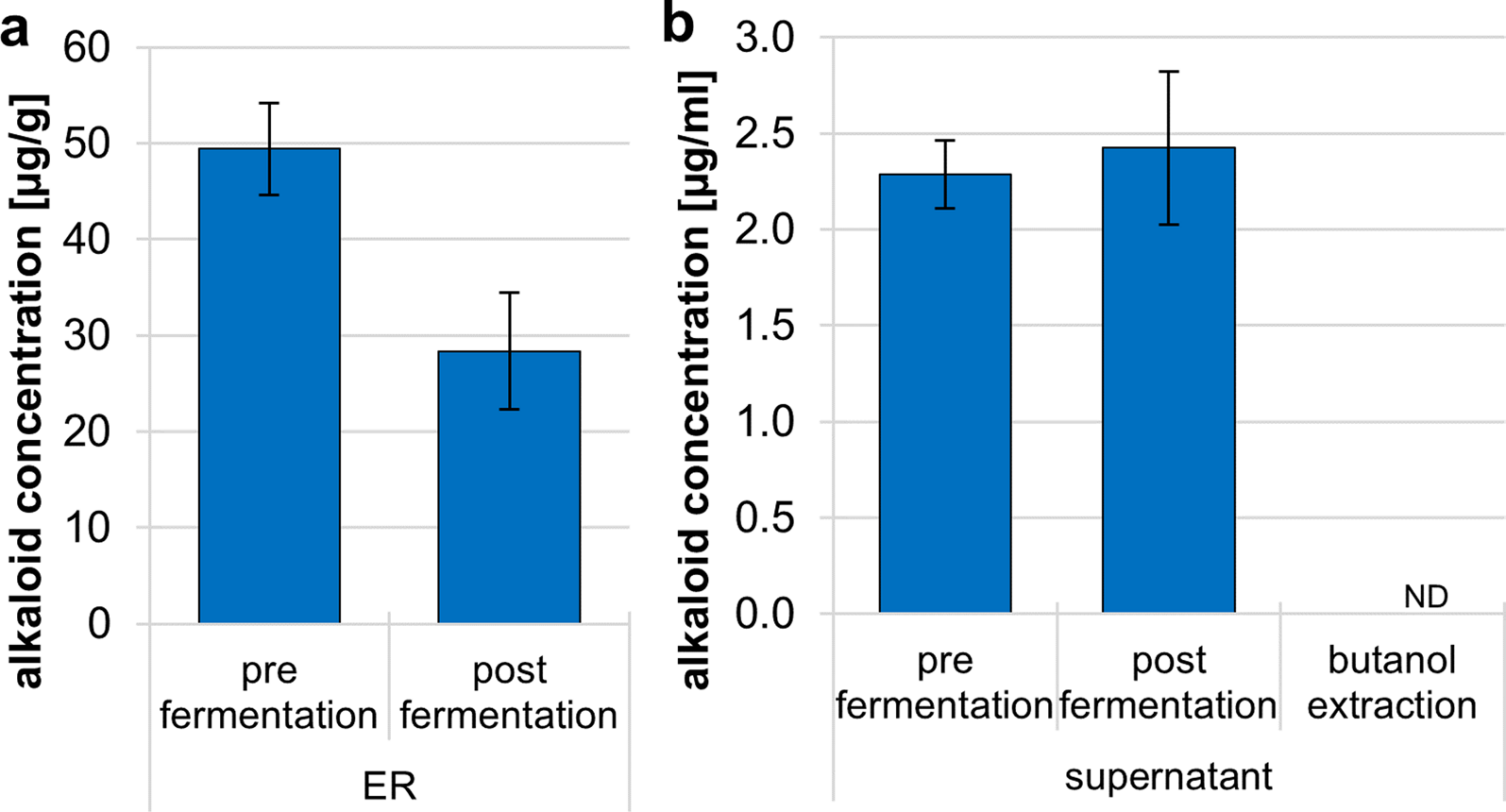
Measurement of ergot alkaloids in ergot contaminated rye and fermentation samples. **a** The ergot alkaloid concentration of pure ER prior and post ABE fermentation with *C. beijerinckii* was determined using the ErgoREAD ELISA kit. The strain was incubated on GM plus 15% ER. **b** The ergot alkaloid content in the culture supernatants before and after ABE fermentation with *C. beijerinckii* was determined as in a. Similarly, the ergot alkaloid concentration in an ABE extraction of culture supernatant was determined. Error bars represent standard deviation (N = 3; N = 2 for ABE extracts). nd, none determined

The ER substrate contains an ergot alkaloid concentration of ~ 49 μg/g biomass, which was reduced to ~ 28 μg/g biomass after fermentation (Fig. 4a). The sterilization of the medium by autoclaving may have contributed to the ergot alkaloid reduction, as intense heat can significantly degrade ergot sclerotia alkaloid content [62]. Pure ergot sclerotia contain an average ergot alkaloid concentration of 659 μg/g, but the concentration in the samples can significantly differ from 1 to 6003 μg/g [23]. As the ER substrate was a mixture of materials (ergot sclerotia, plant residues, small stones, insects, etc.) separated from the milling process, it only contained about 30% of ergot sclerotia.

The culture supernatant prior and post-fermentation with *C. beijerinckii* contained similar ergot alkaloid contents of ~ 2.3 and 2.4 μg/mL (Fig. 4b), respectively. After extraction of the produced ABE from the fermentation broth using rotary evaporation, no ergot alkaloids were detectable in the liquid extract (Fig. 4b), as ergot alkaloids are non-volatile [24]. Extraction methods like distillation or gas-stripping are therefore well suited for product recovery, as they result in solvents without alkaloid contamination. The ergot alkaloids in the fermentation broth or remaining water phase could also be purified this way, which would allow recycling of the water in the fermentation process.

### Semi-continuous ABE fermentation on a WRD plus ER mixture

Based on the results presented so far, we wanted to evaluate if a semi-continuous fermentation of *C. beijerinckii* NCIMB 8052 on WRD plus ER could increase ABE productivity. As the substrates are insoluble, continuous fermentation was deemed not feasible, as tubes would run the risk of clogging and the substrate may settle in the fermenter.

For this experiment, *C. beijerinckii* NCIMB 8052 was incubated in 750 ml GM without l-cysteine at pH 6.5 and with 16.6% (w/v) WRD plus 8.3% (w/v) ER as substrate. The large amount of milling byproducts was chosen to prevent an early abortion of the fermentation due to substrate limitation. For the first 16 h, the fermentation was performed without any medium replacement and 50 rpm stirring. During this period, the pH in the vessel dropped to ~ 5.0 and increased thereafter to a pH of ~ 5.4. At this point, stirring was increased to 200 rpm and new GM medium without carbon source (because the substrate was solid and gelatinized after autoclaving, continuous addition of new substrate was not possible) was pumped into the system. At the same time, spent medium was removed from the vessel. A dilution rate (D) of 0.058 h^−1^ was maintained for the remainder of the fermentation process and samples were taken every 24 h.

**Fig. 5:**
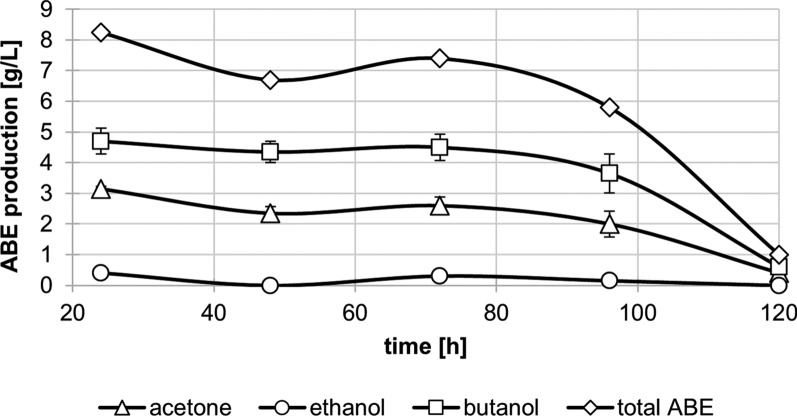
Semi-continuous ABE fermentation of *C. beijerinckii* with WRD and ER as substrate. The strain was incubated in 750 ml GM with 16.6% WRD and 8.3% ER as carbon source. There was no medium replacement for the first 16 h of fermentation and the stirring was set to 50 rpm. Afterwards, stirring was set to 200 rpm and fermentation broth in the fermenter was continuously replaced with sterile new GM medium without carbon source at a D of 0.058 h.^−1^. The fermentation was maintained for 120 h. Samples of the fermentation broth were taken every 24 h. Error bars represent standard deviation (N = 2)

The fermentation process could be maintained for ~ 120 h. At this point, almost no butanol (0.6 g/L) was formed anymore, and the pH had dropped below 5.0. The process was therefore terminated at this point in time. During the first 96 h of fermentation, the average ABE and butanol concentration in the broth reached 7.04 ± 1.04 g/L and 4.30 ± 0.46 g/L, respectively (Fig. 5). The decrease in ABE production in the last 30 h of the fermentation may be explained by nutrient depletion and oxygen diffusion through tubing connectors [63]. Additionally, changes in strain morphology over the course of the fermentation, pointing to stress conditions, may have influenced ABE formation [64]. Especially the onset of sporulation may have reduced solventogenesis, but we did not monitor strain morphology during the course of the fermentation presented here.

A total of ~ 37.7 g ABE containing ~ 25.2 g butanol was produced throughout the fermentation. The process showed an average volumetric ABE productivity during the continuous phase of 0.41 ± 0.07 g L^−1^ h^−1^ for 96 h and ABE productivity per available sugars of 0.37 g g^−1^ (calculated for 100% sugars used). This is already better than in the batch fermentations of *C. beijerinckii* NCIMB 8052 with 10% substrate loading, which achieved a volumetric ABE productivity of only ~ 0.14 g L^−1^ h^−1^ and an ABE yield of 0.17 g/g. The amount of substrate in the fermentation vessel was reduced by ~ 74% (from 168,2 g to 43.2 g dry weight). With a substrate sugar content of approximately 61%, the low residual material suggests significant sugar degradation, reaching at least the batch fermentation level and showing the substrate's good fermentability.

Ezeji et al. could reach volumetric ABE productivities of 1.16 and 0.92 g L^−1^ h^−1^ in fed-batch and continuous fermentations of *C. beijerinckii* BA101 and ABE yield per used sugar of 0.46 und 0.41 g/g, respectively [63, 65]. However, they also performed in situ product recovery by gas-stripping in these fermentations, and additionally, a hyper-producing genetically modified strain *C. beijerinckii* BA101 was used by Ezeji et al*.* [57].

In terms of ABE yield, the process is already comparable with other processes but productivity can be improved [56, 66, 67]. By adopting state-of-the-art in-process product recovery procedures and improved strain derivates could significantly increase ABE production. Targeted genetic engineering of *C. beijerinckii* NCIMB 8052, e.g., down-regulation of CCR and sporulation, should also lead to increased ABE production in this strain.

Still, our data show that efficient butanol production can be achieved by semi-continuous fermentation using milling byproducts as substrate. Milling byproducts (feed wheat ~ 200 €/t and WB 120 €/t on average in the first half of 2024, source: Bavarian Commodities Exchange) are at least twofold cheaper than glucose, which was 430 € in 2019 and exceptional since the beginning of 2024 (~ 650 €/ton; first quarter 2024, source: www.procurementresource.com). As they were well utilized during the semi-continuous fermentation, even slight improvements of the ABE productivity of the process should lead to an economically viable production scale [13].

## Conclusions

In this study, milling byproducts were analyzed as a carbon source for bio-butanol production using clostridial ABE fermentation. The carbohydrate composition of WRD, RSF, WB, RB and ER was determined, which revealed that WRD, RSF, and ER contained significant glucan contents, while WB and RB also contained large amounts of xylan. The glucan portion of all tested milling byproducts is mainly composed of starch, which is a suitable substrate for clostridial fermentation. This was confirmed by ABE fermentation of *C. beijerinckii* NCIMB 8052, *C. diolis* DSM 15410, *C. saccharobutylicum* DSM 13864 and *C. saccharoperbutylacetonicum* N1-504 on these substrates. All strain produced between ~ 7.0 and ~ 13.5 g/L butanol on WRD, RSF and ER. Furthermore, *C. beijerinckii* NCIMB 8052 was also able to produce more than 7 g/L butanol on the bran substrates. The ergot sclerotia-containing waste stream materials can be used to supplement other substrates, as the material alone already led to the production of ~ 9 g/L butanol. ER has no market value and released ergot alkaloids can be removed during butanol extraction without any additional clean-up procedures.

To determine how milling byproducts could be utilized for industrial butanol production, we performed a semi-continuous ABE fermentation of *C. beijerinckii* NCIMB 8052 on a mixture of WRD and ER. The fermentation could be maintained over 96 h and had a productivity in the continuous phase of ~ 0.41 g l^−1^ h^−1^ was achieved, leading to the production of 37.7 g ABE out of 168.2 g substrate. The substrate was utilized to a great extent, as 74% of the WRD plus ER mixture was consumed during the fermentation process. A semi-continuous clostridial ABE fermentation on milling byproducts may therefore be a suitable avenue for commercial butanol production, although the productivity and yield of the process have to be further improved. A feasible approach to achieve these goals might be in situ product recovery and the utilization of genetically engineered clostridia strains.

## Methods

### Clostridia strains and cultivation

The strains *Clostridium beijerinckii* NCIMB 8052, *Clostridium diolis* DSM 15410, *Clostridium saccharobutylicum* DSM 13864 and *Clostridium saccharoperbutylacetonicum* N1-504 were from the strain collection of the Chair of Microbiology (Technical University of Munich, Germany). The strains were stored as spore suspensions.

Cultivation was performed in 20 or 100 ml butyl rubber-stoppered serum bottles, which were made anaerobic by venting the air with vacuum and replacing the headspace with 98% N_2_ + 2% H_2_, followed by autoclaving [68]. The strains were incubated in *Grundmedium* YAF25 (GM), which was composed of 5 g/L yeast extract (Carl Roth, Germany), 65 mM ammonium acetate (Carl Roth), 1 g/L l-cysteine hydrochloride monohydrate (Carl Roth) and 90 µM iron (II) sulfate heptahydrate (Merck, Germany) dissolved in tap water [13]. The pH of the medium was adjusted to 6.8. To activate spores, they were diluted 1:100 in GM with 4% (w/v) sterile filtered d-glucose (Carl Roth) and incubated for 10 min at 60 °C. The spore cultures were incubated for 48 h at 34 °C. Afterwards, the cells were diluted 1:10 in new GM with 4% (w/v) sterile filtered d-glucose and incubated for an additional 24 h. A volume of 5 ml pre-culture was used to inoculate 50 ml GM with wheat red dog (WRD), rye second flour (RSF), wheat bran (WB), rye bran (RB) or ergot sclerotia-containing rye (ER). If substrates were treated with cellic ctec2 (Merck), they were incubated at 50 °C for 24 h with 0.5% (v/v) enzyme mixture prior to inoculation. The strains were incubated for 72 h at 34 °C and culture supernatant samples were taken for gas chromatography (GC).

The substrates were provided by the Bayerischer Müllerbund e.V. (Munich, Germany) and the amount of substrate is listed in the respective experiments. ER was a mixture of rye grains, ergot sclerotia and other unwanted products, such as small stones, insects, and straw, which resulted from optical separation methods during the milling process. The content of ergot sclerotia in these samples was ~ 30%. ER was the only substrate that contained whole grains and therefore had to be milled prior to fermentation (Fig. 1a). This was done using a Retsch PM 100 planetary ball mill.

### Semi-continuous fermentation

A bioreactor system BIOSTAT^®^ B plus (Sartorius, Germany) was used with a 1 l vessel for semi-continuous fermentation. The fermenter contained 750 ml GM without l-cysteine and a mixture of 16.6% (w/v) WRD and 8.3% (w/v) ER was used as carbon source. The pH of the medium was set to 6.5. The vessel was autoclaved at 121 °C for 20 min and made anaerobic by sparging the medium with N_2_ at 65 °C for 30 min. GM without carbon source was prepared the same way and connected to the fermenter as new medium feed. The medium bleed was removed into a separate reservoir bottle. The medium was inoculated with 75 ml of a 24 h old *C. beijerinckii* NCIMB 8052 culture. Prior to inoculation, the fermenter temperature was set to 34 °C and it was sparged with N_2_ for 30 min after inoculation. The culture was incubated for 16 h at 34 °C and 50 rpm stirring, followed by additional 104 h of incubation with continuous medium change and 200 rpm stirring. The dilution rate (D) was set to 0.058 h^−1^. Samples of the fermentation broth were taken daily and measured using GC. The biomass of the remaining substrate after fermentation was transferred to aluminum pans and dried overnight at 105 °C. The mass of the substrate was measured as dry weight.

### Gas chromatography

The concentrations of acetone, ethanol and 1*-*butanol were determined using a Nexis GC 2030 system (Shimadzu, Japan). The samples were diluted 1:10 with distilled water at pH 2 (set with hydrochloric acid) and 0.05% (w/w) 1-propanol was added as internal standard. A volume of 0.5 µl sample was injected at 250 °C on a 30 m Restek Stabilwax^®^-DA column with an inner diameter of 0.32 mm and film thickness of 0.5 µm. The column temperature was set to 40 °C for 5 min. Afterwards a temperature gradient of 15 °C/min from 40 to 100 °C was applied, followed by a gradient of 30 °C/min from 100 to 240 °C. The final temperature was held for 2 min. A flame ionization detector was used to measure the eluted compounds at 260 °C.

### Evaporation of solvents from fermentation broth

A Hei-VAP Core system (Heidolph, Germany) was used to extract solvents from fermentation broth by rotary evaporation according to the manufacturer’s instructions. A volume of 100 ml fermentation broth was poured into a 1-L evaporation flask and attached to the system. The water bath temperature was set to 32 °C and the cooler temperature was set to ~ 10 °C. Vacuum was applied and the evaporation flask was lowered into the water bath. The evaporation process was performed for 10 min at 180 rpm. The extracted solvent–water mixture was collected in a 500-ml collection flask.

### Compositional analysis of substrates

The carbohydrate composition of WRD, wheat bran (WB), rye second flour (RSF), rye bran (RB) and ER was determined as described by [69]. The substrates were dried overnight at 60 °C and analyzed for structural carbohydrates and ash content by using acidic hydrolysis following the National Renewable Energy Laboratory’s (NREL) protocol [70]. Released monosaccharides were measured by high-performance anion exchange chromatography with pulsed amperometric detection (HPAED-PAD) as described previously [49]. The samples were injected into an ICS-3000 instrument (Thermo Scientific, USA) using a 4 mm × 250 mm CarboPac PA1 column (Thermo Scientific) with a 4 mm × 50 mm guard column of the same type (Thermo Scientific). Elution was performed with an isocratic mobile phase of 20 mM sodium hydroxide at 30 °C for 20 min and a flow rate of 1.0 ml/min. Afterwards, the column was equilibrated to 100 mM sodium hydroxide over 3 min, directly followed by a gradient of 0–150 mM sodium acetate in 100 mM sodium hydroxide over 18 min. Ash content of substrates was determined by measuring the dry weight of the remaining biomass after acidic hydrolysis.

The starch content of the substrates was determined using α-amylase from *Aspergillus oryzae* (Merck) and α-glucosidase from *Saccharomyces cerevisiae* (Merck). 10 mg of dry substrate was suspended in 900 µl 50 mM sodium acetate buffer pH 6 containing 1 mM CaCl_2_ and incubated at 80 °C for 60 min with 950 rpm shaking. After cooling down to room temperature, 100 µl of a 500 U α-amylase and 500 U α-glucosidase mixture was added to the substrate suspension. The enzyme reaction was performed for 24 h at 37 °C with agitation at 950 rpm. Full degradation of soluble starch was confirmed by conducting the same experiment with 10 mg soluble starch (Carl Roth) as control. Released monosaccharides were determined using a modified version of the Megazyme *p*-hydroxybenzoic acid hydrazide (PAHBAH) reducing end assay [71].

### Determination of the ergot alkaloid concentration

The ergot alkaloid content of ER was determined using the ELISA *Schnelltest* ErgoREAD (LCTech, Germany) according to the manufacturer’s instructions. Additionally, the ergot alkaloid concentration of culture supernatant and the solvent–water mixture extracted by rotary evaporation were measured using the same kit.

## Supplementary Information

Additional file 1: Fig. S1: Calculation of product yields for the degradation experiment of figure 3. a) Calculation of product yield per total fermentation substrate. b) Calculation of product yield per used sugar. Values represent the mean of three biological replicates.

Additional file 2: Table S 1 – Analyzed values for solvent production for the degradation experiment of figure 3.

## Data Availability

No datasets were generated or analysed during the current study.

## Abbreviations

ER: Ergot sclerotia-containing rye
GC: Gas chromatography
GM: *Grundmedium* YAF25
HPAEC-PAD: High-performance anion exchange chromatography with pulsed amperometric detection
NREL: National Renewable Energy Laboratory
PAHBAH: *p*-Hydroxybenzoic acid hydrazide
RB: Rye bran
RSF: Rye second flour
TUM: Technical University of Munich
WB: Wheat bran
WRD: Wheat red dog

## References

[1] Pachauri R RA. Climate change 2007: synthesis report: Contribution of working groups I, II and III to the fourth Assessment report of the Intergovernmental Panel on Climate Change. 2007.

[2] Liao JC, Mi L, Pontrelli S, Luo S. Fuelling the future: microbial engineering for the production of sustainable biofuels. Nat Rev Microbiol. 2016;14:288–304. 10.1038/nrmicro.2016.32. 27026253 10.1038/nrmicro.2016.32

[3] Carroll A, Somerville C. Cellulosic biofuels. Annu Rev Plant Biol. 2009;60:165–82. 10.1146/annurev.arplant.043008.092125. 19014348 10.1146/annurev.arplant.043008.092125

[4] Dürre P. Biobutanol: an attractive biofuel. Biotechnol J. 2007;2:1525–34. 10.1002/biot.200700168. 17924389 10.1002/biot.200700168

[5] López-Contreras AM, Kuit W, Springer J. Novel Strategies for Production of Medium and High Chain Length Alcohols. In: Hallenbeck PC, editor. Microbial Technologies in Advanced Biofuels Production. Springer, US: Boston MA; 2012. p. 183–211.

[6] Xue C, Zhao X-Q, Liu C-G, Chen L-J, Bai F-W. Prospective and development of butanol as an advanced biofuel. Biotechnol Adv. 2013;31:1575–84. 10.1016/j.biotechadv.2013.08.004. 23993946 10.1016/j.biotechadv.2013.08.004

[7] Galloni E, Fontana G, Staccone S, Scala F. Performance analyses of a spark-ignition engine firing with gasoline–butanol blends at partial load operation. Energy Convers Manage. 2016;110:319–26. 10.1016/j.enconman.2015.12.038.

[8] Yun H, Choi K, Lee CS. Effects of biobutanol and biobutanol–diesel blends on combustion and emission characteristics in a passenger car diesel engine with pilot injection strategies. Energy Convers Manage. 2016;111:79–88. 10.1016/j.enconman.2015.12.017.

[9] Lee SY, Park JH, Jang SH, Nielsen LK, Kim J, Jung KS. Fermentative butanol production by Clostridia. Biotechnol Bioeng. 2008;101:209–28. 10.1002/bit.22003. 18727018 10.1002/bit.22003

[10] Zverlov VV, Berezina O, Velikodvorskaya GA, Schwarz WH. Bacterial acetone and butanol production by industrial fermentation in the Soviet Union: use of hydrolyzed agricultural waste for biorefinery. Appl Microbiol Biotechnol. 2006;71:587–97. 10.1007/s00253-006-0445-z. 16685494 10.1007/s00253-006-0445-z

[11] Ezeji T, Qureshi N, Blaschek HP. Production of acetone–butanol–ethanol (ABE) in a continuous flow bioreactor using degermed corn and Clostridium beijerinckii. Process Biochem. 2007;42:34–9. 10.1016/j.procbio.2006.07.020.

[12] Al-Shorgani NKN, Al-Tabib AI, Kadier A, Zanil MF, Lee KM, Kalil MS. Continuous butanol fermentation of dilute acid-pretreated de-oiled rice bran by clostridium acetobutylicum YM1. Sci Rep. 2019;9:4622. 10.1038/s41598-019-40840-y. 30874578 10.1038/s41598-019-40840-y PMC6420626

[13] Thieme N, Panitz JC, Held C, Lewandowski B, Schwarz WH, Liebl W, Zverlov V. Milling byproducts are an economically viable substrate for butanol production using clostridial ABE fermentation. Appl Microbiol Biotechnol. 2020;104:8679–89. 10.1007/s00253-020-10882-8. 32915256 10.1007/s00253-020-10882-8 PMC7502454

[14] Huang Q, Shi CX, Su YB, Liu ZY, Li DF, Liu L, et al. Prediction of the digestible and metabolizable energy content of wheat milling by-products for growing pigs from chemical composition. Anim Feed Sci Technol. 2014;196:107–16. 10.1016/j.anifeedsci.2014.06.009.

[15] Vogel J. Unique aspects of the grass cell wall. Curr Opin Plant Biol. 2008;11:301–7. 10.1016/j.pbi.2008.03.002. 18434239 10.1016/j.pbi.2008.03.002

[16] Bayerische Landesanstalt für Landwirtschaft. Nebenerzeugnisse der Mehlmüllerei: Ergebnisse eines Projektes zur Untersuchung des Futterwertes von Mühlennachprodukten. 2012.

[17] Berezina OV, Sineoky SP, Velikodvorskaya GA, Schwarz W, Zverlov VV. Extracellular glycosyl hydrolase activity of the Clostridium strains producing acetone, butanol, and ethanol. Appl Biochem Microbiol. 2008;44:42–7. 10.1134/s0003683808010079.

[18] Berezina OV, Zakharova NV, Yarotsky CV, Zverlov VV. Microbial producers of butanol. Appl Biochem Microbiol. 2012;48:625–38. 10.1134/S0003683812070022.

[19] Zhang Y-HP. Production of biofuels and biochemicals by in vitro synthetic biosystems: opportunities and challenges. Biotechnol Adv. 2015;33:1467–83. 10.1016/j.biotechadv.2014.10.009. 25447781 10.1016/j.biotechadv.2014.10.009

[20] Tudzynski P, Hölter K, Correia T, Arntz C, Grammel N, Keller U. Evidence for an ergot alkaloid gene cluster in Claviceps purpurea. Mol Gen Genet. 1999;261:133–41. 10.1007/S004380050950. 10071219 10.1007/s004380050950

[21] Hulvová H, Galuszka P, Frébortová J, Frébort I. Parasitic fungus Claviceps as a source for biotechnological production of ergot alkaloids. Biotechnol Adv. 2013;31:79–89. 10.1016/j.biotechadv.2012.01.005. 22261014 10.1016/j.biotechadv.2012.01.005

[22] Agriopoulou S. Ergot alkaloids mycotoxins in cereals and cereal-derived food products: characteristics, toxicity, prevalence, and control strategies. Agronomy. 2021;11:931. 10.3390/agronomy11050931.

[23] Mulder P, van Raamsdonk L, van Egmond HJ, Voogt, Van Brakel, J. M.W. Dutch survey ergot alkaloids and sclerotia in animal feeds. 2012.

[24] Schiff PL. Ergot and its alkaloids. Am J Pharm Educ. 2006;70:98. 10.5688/aj700598. 17149427 10.5688/aj700598 PMC1637017

[25] Haarmann T, Rolke Y, Giesbert S, Tudzynski P. Ergot: from witchcraft to biotechnology. Mol Plant Pathol. 2009;10:563–77. 10.1111/J.1364-3703.2009.00548.X. 19523108 10.1111/j.1364-3703.2009.00548.x PMC6640538

[26] Campbell WP. Infection of barley by claviceps purpurea. Can J Bot. 1958;36:615–9. 10.1139/b58-057.

[27] Corbett K, Dickerson AG, Mantle PG. Metabolic studies on Claviceps purpurea during parasitic development on rye. J Gen Microbiol. 1974;84:39–58. 10.1099/00221287-84-1-39. 4436648 10.1099/00221287-84-1-39

[28] Mantle PG, Shaw S. Role of ascospore production by Claviceps purpurea in aetiology of ergot disease in male sterile wheat. Trans Br Mycol Soc. 1976;67:17–22. 10.1016/S0007-1536(76)80002-2.

[29] Miedaner T, Geiger HH. Biology, genetics, and management of ergot (Claviceps spp.) in rye, sorghum, and pearl millet. Toxins. 2015;7:659–78. 10.3390/toxins7030659. 25723323 10.3390/toxins7030659 PMC4379517

[30] Bandyopadhyay R, Frederickson DE, McLaren NW, Odvody GN, Ryley MJ. Ergot: a new disease threat to sorghum in the Americas and Australia. Plant Dis. 1998;82:356–67. 10.1094/PDIS.1998.82.4.356. 30856881 10.1094/PDIS.1998.82.4.356

[31] Knudsen KEB. Carbohydrate and lignin contents of plant materials used in animal feeding. Anim Feed Sci Technol. 1997;67:319–38. 10.1016/S0377-8401(97)00009-6.

[32] Salo M-L, Kotilainen K. On the carbohydrate composition and nutritive value of some cereals. AFSci. 1970;42:21–9. 10.23986/afsci.71752.

[33] Nandini CD, Salimath PV. Carbohydrate composition of wheat, wheat bran, sorghum and bajra with good chapati/roti (Indian flat bread) making quality. Food Chem. 2001;73:197–203. 10.1016/S0308-8146(00)00278-8.

[34] Green MM, Blankenhorn G, Hart H. Which starch fraction is water-soluble, amylose or amylopectin? J Chem Educ. 1975;52:729. 10.1021/ed052p729.

[35] Zhong F, Yokoyama W, Wang Q, Shoemaker CF. Rice starch, amylopectin, and amylose: molecular weight and solubility in dimethyl sulfoxide-based solvents. J Agric Food Chem. 2006;54:2320–6. 10.1021/jf051918i. 16536614 10.1021/jf051918i

[36] Juhász R, Salgó A. Pasting behavior of amylose, amylopectin and their mixtures as determined by RVA curves and first derivatives. Starch Stärke. 2008;60:70–8. 10.1002/star.200700634.

[37] Marc A, Engasser JM, Moll M, Flayeux R. A kinetic model of starch hydrolysis by alpha- and beta-amylase during mashing. Biotechnol Bioeng. 1983;25:481–96. 10.1002/bit.260250214. 18548665 10.1002/bit.260250214

[38] Ounine K, Petitdemange H, Raval G, Gay R. Regulation and butanol inhibition of D-xylose and D-glucose uptake in Clostridium acetobutylicum. Appl Environ Microbiol. 1985;49:874–8. 10.1128/AEM.49.4.874-878.1985. 4004220 10.1128/aem.49.4.874-878.1985 PMC238462

[39] Gancedo JM. Yeast carbon catabolite repression. Microbiol Mol Biol Rev. 1998;62:334–61. 10.1128/MMBR.62.2.334-361.1998. 9618445 10.1128/mmbr.62.2.334-361.1998 PMC98918

[40] Stülke J, Hillen W. Carbon catabolite repression in bacteria. Curr Opin Microbiol. 1999;2:195–201. 10.1016/S1369-5274(99)80034-4. 10322165 10.1016/S1369-5274(99)80034-4

[41] Pauly M, Keegstra K. Cell-wall carbohydrates and their modification as a resource for biofuels. Plant J. 2008;54:559–68. 10.1111/j.1365-313X.2008.03463.x. 18476863 10.1111/j.1365-313X.2008.03463.x

[42] Han SO, Yukawa H, Inui M, Doi RH. Regulation of expression of cellulosomal cellulase and hemicellulase genes in Clostridium cellulovorans. J Bacteriol. 2003;185:6067–75. 10.1128/JB.185.20.6067-6075.2003. 14526018 10.1128/JB.185.20.6067-6075.2003 PMC225016

[43] Broeker J, Mechelke M, Baudrexl M, Mennerich D, Hornburg D, Mann M, et al. The hemicellulose-degrading enzyme system of the thermophilic bacterium Clostridium stercorarium: comparative characterisation and addition of new hemicellulolytic glycoside hydrolases. Biotechnol Biofuels. 2018;11:229. 10.1186/s13068-018-1228-3. 30159029 10.1186/s13068-018-1228-3 PMC6106730

[44] Song Y, Xue Y, Ma Y. Global microarray analysis of carbohydrate use in alkaliphilic hemicellulolytic bacterium Bacillus sp N16–5. PLoS ONE. 2013. 10.1371/journal.pone.0054090. 23326578 10.1371/journal.pone.0054090 PMC3542313

[45] Kumar P, Barrett DM, Delwiche MJ, Stroeve P. Methods for Pretreatment of lignocellulosic biomass for efficient hydrolysis and biofuel production. Ind Eng Chem Res. 2009;48:3713–29. 10.1021/ie801542g.

[46] Wyman CE, Dale BE, Elander RT, Holtzapple M, Ladisch MR, Lee YY. Coordinated development of leading biomass pretreatment technologies. Bioresour Technol. 2005;96:1959–66. 10.1016/j.biortech.2005.01.010. 16112483 10.1016/j.biortech.2005.01.010

[47] Brandt A, Ray MJ, To TQ, Leak DJ, Murphy RJ, Welton T. Ionic liquid pretreatment of lignocellulosic biomass with ionic liquid–water mixtures. Green Chem. 2011;13:2489. 10.1039/c1gc15374a.

[48] Brandt A, Gräsvik J, Hallett JP, Welton T. Deconstruction of lignocellulosic biomass with ionic liquids. Green Chem. 2013;15:550. 10.1039/c2gc36364j.

[49] Isci A, Thieme N, Lamp A, Zverlov V, Kaltschmitt M. Production of xylo-oligosaccharides from wheat straw using microwave assisted deep eutectic solvent pretreatment. Ind Crops Prod. 2021;164: 113393. 10.1016/J.INDCROP.2021.113393.

[50] Hosseini Koupaie E, Dahadha S, Bazyar Lakeh AA, Azizi A, Elbeshbishy E. Enzymatic pretreatment of lignocellulosic biomass for enhanced biomethane production-a review. J Environ Manage. 2019;233:774–84. 10.1016/j.jenvman.2018.09.106. 30314871 10.1016/j.jenvman.2018.09.106

[51] Englesberg E. Glucose inhibition and the Diauxie phenomenon. Proc Natl Acad Sci U S A. 1959;45:1494–507. 10.1073/pnas.45.10.1494. 16590532 10.1073/pnas.45.10.1494 PMC222744

[52] Buendia-Kandia F, Rondags E, Framboisier X, Mauviel G, Dufour A, Guedon E. Diauxic growth of Clostridium acetobutylicum ATCC 824 when grown on mixtures of glucose and cellobiose. AMB Express. 2018;8:85. 10.1186/s13568-018-0615-2. 29789978 10.1186/s13568-018-0615-2 PMC5964051

[53] Patakova P, Linhova M, Rychtera M, Paulova L, Melzoch K. Novel and neglected issues of acetone–butanol–ethanol (ABE) fermentation by clostridia: clostridium metabolic diversity, tools for process mapping and continuous fermentation systems. Biotechnol Adv. 2013;31:58–67. 10.1016/j.biotechadv.2012.01.010. 22306328 10.1016/j.biotechadv.2012.01.010

[54] Shaheen R, Shirley M, Jones DT. Comparative fermentation studies of industrial strains belonging to four species of solvent-producing clostridia. J Mol Microbiol Biotechnol. 2000;2:115–24. 10937496

[55] Jones DT, Schulz F, Roux S, Brown SD. Solvent-producing clostridia revisited. Microorganisms. 2023. 10.3390/microorganisms11092253. 37764097 10.3390/microorganisms11092253 PMC10538166

[56] Nguyen N-P-T, Raynaud C, Meynial-Salles I, Soucaille P. Reviving the Weizmann process for commercial n-butanol production. Nat Commun. 2018;9:3682. 10.1038/s41467-018-05661-z. 30206218 10.1038/s41467-018-05661-z PMC6134114

[57] Qureshi N, Blaschek HP. Recent advances in ABE fermentation: hyper-butanol producing Clostridium beijerinckii BA101. J Ind Microbiol Biotechnol. 2001;27:287–91. 10.1038/sj.jim.7000114. 11781803 10.1038/sj.jim.7000114

[58] Liu S, Qureshi N. How microbes tolerate ethanol and butanol. N Biotechnol. 2009;26:117–21. 10.1016/j.nbt.2009.06.984. 19577017 10.1016/j.nbt.2009.06.984

[59] Ezeji T, Milne C, Price ND, Blaschek HP. Achievements and perspectives to overcome the poor solvent resistance in acetone and butanol-producing microorganisms. Appl Microbiol Biotechnol. 2010;85:1697–712. 10.1007/s00253-009-2390-0. 20033401 10.1007/s00253-009-2390-0

[60] Madihah MS, Ariff AB, Sahaid KM, Suraini AA, Karim M. Direct fermentation of gelatinized sago starch to acetone–butanol–ethanol by Clostridium acetobutylicum. World J Microbiol Biotechnol. 2001;17:567–76. 10.1023/A:1012351112351.

[61] Li S-Y, Srivastava R, Suib SL, Li Y, Parnas RS. Performance of batch, fed-batch, and continuous A-B-E fermentation with pH-control. Bioresour Technol. 2011;102:4241–50. 10.1016/j.biortech.2010.12.078. 21227684 10.1016/j.biortech.2010.12.078

[62] Young JC, Chen ZJ, Marquardt RR. Reduction in alkaloid content of ergot sclerotia by chemical and physical treatment. J Agric Food Chem. 1983;31:413–5. 10.1021/jf00116a057. 6853863 10.1021/jf00116a057

[63] Ezeji TC, Qureshi N, Blaschek HP. Acetone butanol ethanol (ABE) production from concentrated substrate: reduction in substrate inhibition by fed-batch technique and product inhibition by gas stripping. Appl Microbiol Biotechnol. 2004;63:653–8. 10.1007/s00253-003-1400-x. 12910325 10.1007/s00253-003-1400-x

[64] Qureshi N, Paterson AHJ, Maddox IS. Model for continuous production of solvents from whey permeate in a packed bed reactor using cells of Clostridium acetobutylicum immobilized by adsorption onto bonechar. Appl Microbiol Biotechnol. 1988;29:323–8. 10.1007/BF00265814.

[65] Ezeji TC, Qureshi N, Blaschek HP. Microbial production of a biofuel (acetone–butanol–ethanol) in a continuous bioreactor: impact of bleed and simultaneous product removal. Bioprocess Biosyst Eng. 2013;36:109–16. 10.1007/s00449-012-0766-5. 22729675 10.1007/s00449-012-0766-5

[66] Xue C, Zhao J-B, Chen L-J, Bai F-W, Yang S-T, Sun J-X. Integrated butanol recovery for an advanced biofuel: current state and prospects. Appl Microbiol Biotechnol. 2014;98:3463–74. 10.1007/s00253-014-5561-6. 24535254 10.1007/s00253-014-5561-6

[67] Outram V, Lalander C-A, Lee JGM, Davies ET, Harvey AP. Applied in situ product recovery in ABE fermentation. Biotechnol Prog. 2017;33:563–79. 10.1002/btpr.2446. 28188696 10.1002/btpr.2446 PMC5485034

[68] Rettenmaier R, Gerbaulet M, Liebl W, Zverlov VV. Hungateiclostridium mesophilum sp. Nov., a mesophilic, cellulolytic and spore-forming bacterium isolated from a biogas fermenter fed with maize silage. Int J Syst Evol Microbiol. 2019;69:3567–73. 10.1099/ijsem.0.003663. 31429816 10.1099/ijsem.0.003663

[69] Benz JP, Chau BH, Zheng D, Bauer S, Glass NL, Somerville CR. A comparative systems analysis of polysaccharide-elicited responses in neurospora crassa reveals carbon source-specific cellular adaptations. Mol Microbiol. 2014;91:275–99. 10.1111/mmi.12459. 24224966 10.1111/mmi.12459 PMC3900418

[70] National Renewable Energy Laboratory (NREL). NREL is a national laboratory of the U.S. Department of Energy, Office of Energy Efficiency & Renewable Energy, operated by the Alliance for Sustainable Energy, LLC. National Renewable Energy Laboratory 15013 Denver West Parkway Golden, Colorado 80401 303–275–3000 • www.nrel.gov Contract No. DE-AC36–08GO28308 Determination of Structural Carbohydrates and Lignin in Biomass: Laboratory Analytical Procedure (LAP). 2012. http://www.nrel.gov/biomass/analytical_procedures.html.

[71] Powell JC, Lever M. A new automated procedure for the colorimetric determination of glucose. Biochem Med. 1972;6:543–7. 10.1016/0006-2944(72)90008-7. 4640191 10.1016/0006-2944(72)90008-7

