# Supplementary figures and images for: Valorization of milling byproducts and ergot-sclerotia-contaminated rye via clostridial ABE fermentation
Source: Biotechnol Biofuels Bioprod. 2024 Nov 30;17:139. doi: 10.1186/s13068-024-02590-6 (PMC11607984; doi:10.1186/s13068-024-02590-6)

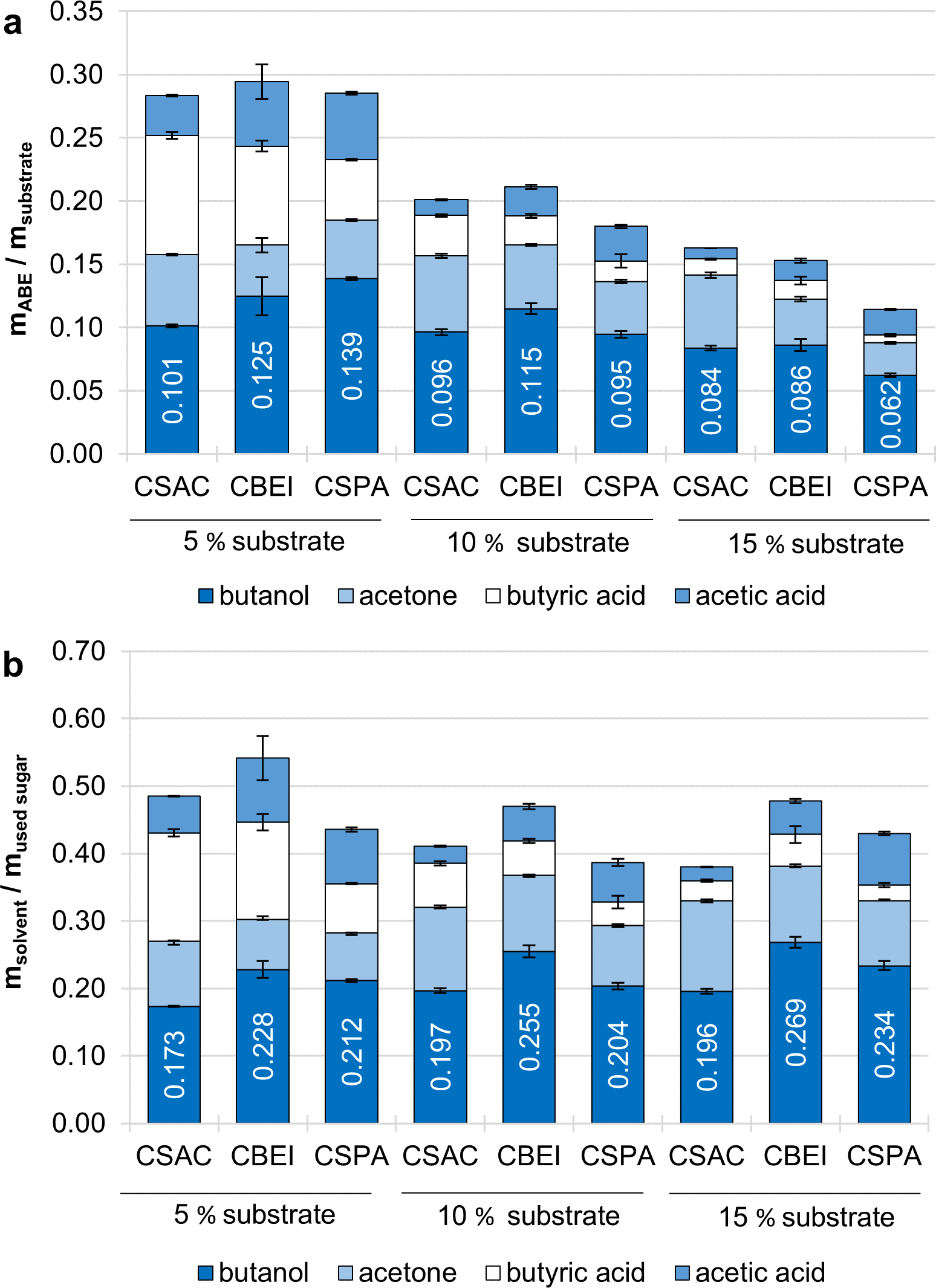

Supplement: Supplementary file 1 — Additional file 1: Fig. S1: Calculation of product yields for the degradation experiment of figure 3. a) Calculation of product yield per total fermentation substrate. b) Calculation of product yield per used sugar. Values represent the mean of three biological replicates. [file 13068_2024_2590_MOESM1_ESM.png]
