# Supplementary material for: Valorization of milling byproducts and ergot-sclerotia-contaminated rye via clostridial ABE fermentation
Source: Biotechnol Biofuels Bioprod. 2024 Nov 30;17:139. doi: 10.1186/s13068-024-02590-6 (PMC11607984; doi:10.1186/s13068-024-02590-6)
Supplement: Supplementary file 2 — Additional file 2: Table S 1 – Analyzed values for solvent production for the degradation experiment of figure 3. [file 13068_2024_2590_MOESM2_ESM.docx]

**Fig. S1: Calculation of product yields for the degradation experiment of figure 3. a)** Calculation of product yield per total fermentation substrate. **b)** Calculation of product yield per used sugar. Values represent the mean of three biological replicates (N=3).

**Table S 1 – Analysed values for solvent production for the degradation experiment of figure 3.**

| **substrate** | **strain** | **butanol** | | **acetone** | | **butyric acid** | | **acetic acid** | |
| --- | --- | --- | --- | --- | --- | --- | --- | --- | --- |
|  |  | **mean** | **sd** | **mean** | **sd** | **mean** | **sd** | **mean** | **sd** |
| **5 %** | **Blank** | 0.00 | ±0.00 | 0.00 | ±0.00 | 0.05 | ±0.07 | 4.35 | ±0.07 |
| **5 %** | **CSAC** | 5.07 | ±0.06 | 2.82 | ±0.03 | 4.70 | ±0.13 | 1.58 | ±0.03 |
| **5 %** | **CBEI** | 6.24 | ±0.75 | 2.03 | ±0.29 | 3.91 | ±0.21 | 2.54 | ±0.68 |
| **5 %** | **CSPA** | 6.74 | ±0.39 | 2.25 | ±0.14 | 2.39 | ±0.03 | 2.59 | ±0.10 |
| **10 %** | **CSAC** | 9.63 | ±0.25 | 6.03 | ±0.15 | 3.20 | ±0.10 | 1.23 | ±0.06 |
| **10 %** | **CBEI** | 11.48 | ±0.43 | 5.05 | ±0.06 | 2.30 | ±0.16 | 2.30 | ±0.18 |
| **10 %** | **CSPA** | 9.25 | ±0.54 | 4.03 | ±0.38 | 1.83 | ±0.64 | 2.43 | ±0.35 |
| **15 %** | **CSAC** | 12.57 | ±0.29 | 8.63 | ±0.31 | 1.93 | ±0.06 | 1.30 | ±0.00 |
| **15 %** | **CBEI** | 12.93 | ±0.71 | 5.43 | ±0.29 | 2.23 | ±0.45 | 2.38 | ±0.25 |
| **15 %** | **CSPA** | 9.33 | ±0.21 | 3.83 | ±0.12 | 0.93 | ±0.15 | 3.07 | ±0.06 |
